# Male Partner Involvement in Child Feeding and Associated Factors Among Children Aged 6 to 23 Months in Hawassa City, Sidama Region, Southern Ethiopia: A Community Based Cross Sectional Study

**DOI:** 10.1002/fsn3.70557

**Published:** 2025-07-01

**Authors:** Amelo Bolka, Mahlet Assaye, Assefa Phliphos

**Affiliations:** ^1^ School of Public Health Yirgalem Hospital Medical College Yirgalem Ethiopia

**Keywords:** child feeding, children aged 6–23 months, Hawassa city, male partner involvement, Sidama region

## Abstract

Suboptimal child feeding practices significantly contribute to malnutrition. While maternal involvement in feeding children aged 6–23 months is well‐documented, evidence on male partner engagement remains limited. This study aimed to assess male partner involvement in child feeding and identify associated factors in Hawassa City, Sidama Region. A community‐based cross‐sectional study was conducted from February 1–30, 2024, among 422 male partners with children aged 6–23 months. Participants were selected using systematic sampling. Data were collected via structured, pretested, interviewer‐administered questionnaires. Data analysis was done using SPSS (Statistical Package for the Social Sciences) version 26. Associated factors of male involvement in child feeding were identified using a logistic regression model, and the outputs were presented using adjusted odds ratio (AOR) with a 95% confidence interval (CI). The prevalence of good male partner involvement was 68.7% (95% CI: 64.7–72.7). Factors significantly associated with involvement included: age ≤ 25 years (AOR = 2.32, 95% CI: 1.07–5.31), age 26–35 years (AOR = 2.38, 95% CI: 1.39–4.09), no formal education (AOR = 0.28, 95% CI: 0.14–0.59), child age 6–11 months (AOR = 1.94, 95% CI: 1.13–3.32), good knowledge (AOR = 2.14, 95% CI: 1.26–3.64), positive attitude (AOR = 2.66, 95% CI: 1.55–4.56), and belief in community gender‐specific role norms (AOR = 0.41, 95% CI: 0.24–0.69). Male partner involvement in child feeding was relatively high in Hawassa City. Younger age, lack of formal education, child age, good knowledge, positive attitudes, and gender norms were significant factors. Enhancing knowledge, attitudes, and addressing gender norms could further improve male engagement in child feeding.

## Graphical abstract

This study assessed male partner involvement in child feeding and associated factors among 422 households in Hawassa City, Ethiopia. Using a community‐based cross‐sectional design, the study found 68.7% prevalence of good involvement, with significant associations linked to age, education, child age, knowledge, attitudes, and gender norms. Interventions targeting knowledge, attitudes, and gender norms are recommended to enhance male engagement in child feeding practices.

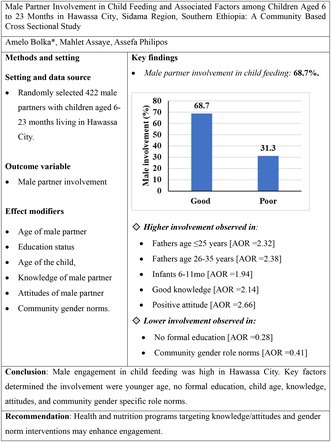

**Abbreviations**
ANC: Antenatal care
AOR: Adjusted odds ratio
BF: Breast feeding
CF: Complementary feeding
CHIS: Community health information system
CI: Confidence interval
CL: Confidence level
COR: Crude odds ratio
DD: Dietary diversity
EBF: Exclusive breastfeeding
EDHS: Ethiopia Demographic and Health Survey
FAO: Food and Agriculture Organization
HH: Household
IQR: Interquartile range
IRB: Institutional Review Board
IYCF: Infant young child feeding
MDD: Minimum dietary diversity
MOH: Ministry of Health
NNP: National Nutrition Program
SD: Standard deviation
SDG: Sustainable Development Goal
SPSS: Statistical Package Software for Statistics
UNICEF: United Nations International Children's Emergency Fund
WFP: World Food Program
WHO: World Health Organization

## 1 Background

Child malnutrition remains a pressing global health challenge, disproportionately impacting children under 5 years of age, particularly in low‐ and middle‐income nations (UNICEF and WHO 2021). Globally, more than 2.7 million children in this age group lose their lives, while 149 million suffer from stunting, 45 million from wasting, and 38.9 million from overweight or obesity (World Health Organization 2020). These figures highlight the profound implications of insufficient nutrition during the critical first 1000 days of life, a period during which nutritional deficiencies can result in enduring deficits in cognitive function, academic performance, economic productivity, and general well‐being (Black et al. 2013; Grantham‐McGregor et al. 2007).

Suboptimal feeding practices for infants and young children play a major role in driving childhood malnutrition. These practices include insufficient breastfeeding, the introduction of inappropriate foods, and feeding methods that do not align with the nutritional requirements of young children (WHO 2020). Although advancements have been made in addressing child nutrition, poor infant and young child feeding (IYCF) practices remain widespread, particularly in low‐ and middle‐income countries. This has led to approximately 159 million children under five experiencing underweight or stunting (Dasgupta et al. 2014). As a result, implementing strategies such as nutrition education, promoting exclusive breastfeeding, improving complementary feeding practices, and fostering behavior change is critical for reducing the prevalence of childhood malnutrition (Habtewold et al. 2017).

Recent studies highlight the influential role of fathers in child nutrition, emphasizing their substantial contribution to both maternal and child health outcomes (Sarkadi et al. 2008). Despite this, deeply ingrained cultural expectations and traditional gender roles frequently hinder paternal engagement, as child feeding is often regarded as the mother's primary duty (Bilal 2015; Etowa et al. 2022). Nevertheless, fathers can play a pivotal role by actively participating in feeding routines, making well‐informed dietary choices, offering financial and emotional support, and fostering open discussions about their child's nutritional requirements (Apriyanto 2020; Moura and Philippe 2023). Moreover, they can encourage healthy eating behaviors by modeling positive dietary habits and contributing to the creation of a supportive and nutritionally enriching home environment (Etowa et al. 2022).

Evidence indicates that the active participation of male partners in child nutrition is linked to enhanced growth, development, and a lower risk of illness and mortality in young children (Apriyanto 2020; Kansiime et al. 2017). A study conducted in Ethiopia found that dietary diversity among children aged 6–23 months increased by 13.7%, highlighting the beneficial effects of paternal involvement on nutritional outcomes (Dafursa and Gebremedhin 2019). Moreover, male partners engagement in feeding practices has been associated with fostering children's social and emotional development, reinforcing the need to integrate male partners into child nutrition strategies (Sarkadi et al. 2008).

Recognizing the critical role of males in child nutrition, public health initiatives increasingly advocate for the active engagement of men in supporting maternal, infant, and young child nutrition (Matovu et al. 2008). Strengthening male participation is not only vital for enhancing child dietary outcomes but also contributes to fostering healthier family relationships and advancing gender equality within households (Berti and Socha 2023). Amid ongoing challenges in IYCF practices, especially in Ethiopia, understanding the extent of male partner participation in child feeding is crucial for designing targeted and impactful interventions (Mekonnen et al. 2018).

Many studies have assessed maternal roles in feeding practices for children aged 6–23 months (Kassa et al. 2016; Fikadu and Girma 2018; Fentahun et al. 2020; Fentaw Mulaw et al. 2020), yet information on male partners' involvement remains scarce. Thus, this study aimed to assess male partner involvement in child feeding and associated factors among children aged 6–23 months in Hawassa City Administration, Sidama Region.

## 2 Methods

### 2.1 Study Setting

This study was conducted in Hawassa City, the capital of Sidama National Regional State located 273 kilometers south of Addis Ababa, the capital of Ethiopia. Based on the 2007 Ethiopia Central Statistical Agency report estimation, the total population of the city administration was projected to be 402,903, of which 14,141 were children aged 6–23 months (Hawassa City Administration Health Department 2023).

### 2.2 Study Design and Period

A community‐based cross‐sectional study design was carried out from 1 to 30 February 2024.

#### 2.2.1 Study Population and Their Eligibility Criteria

Male partners randomly selected from households which had children aged 6–23 months living in the selected kebeles of Hawassa city were the study population. Households with lactating women within 6 months postpartum or without a male partner were excluded.

### 2.3 Sample Size Determination

The sample size adequate for estimating the prevalence of male partner involvement was calculated using a single population proportion formula. The assumptions considered were a 95% confidence level, an expected prevalence of male involvement of 50.9% (Wolkanto et al. 2023), and a 10% non‐response rate. Ultimately, a sample size of 422 was computed.

### 2.4 Sampling Procedure

Out of the 32 kebeles (the smallest administrative units in Ethiopia) within the city administration, nine were chosen through simple random sampling. The sample size was distributed proportionally according to the number of households in each selected kebele that included children aged 6–23 months. Households meeting this criterion were identified using systematic random sampling, based on administrative records maintained by urban health extension workers. In cases where multiple male partners were present in a single household, one individual was randomly selected using a lottery‐based approach.

### 2.5 Data Collection

Data were collected by trained data collectors and supervisors using a structured, pretested, and interviewer‐administered questionnaire. The questionnaire was developed based on the objectives of the study after thoroughly reviewing related literature (Wolkanto et al. 2023; CSAE 2019; Bogale et al. 2022). The tool was initially developed in English and translated into Amharic by a proficient bilingual professional. It was subsequently back‐translated into English by another language expert. Then, the questionnaire was uploaded on KoboToolbox for electronic data collection.

Male involvement in child feeding was assessed using a 22‐item questionnaire with binary (yes/no) responses, covering five domains: shared decision‐making (6 items), physical support to mothers (5 items), psychosocial support (3 items), financial and resource support (5 items), and sharing feeding responsibilities (3 items). Each “yes” response scored 1, indicating participation, while “no” responses scored 0, indicating non‐participation. Total scores (range: 0–22) reflected involvement level, with ≥ 18 (≥ 80%) indicating good involvement and < 18 poor involvement (Bogale et al. 2022; Catholic Relief Services 2016).

Male partners' knowledge of child feeding was measured using a 9‐item questionnaire, with each correct answer scored as 1 and incorrect answers as 0. The total knowledge score was determined by summing correct responses (Bogale et al. 2022). Participants were classified as having good knowledge if they scored ≥ 7 (≥ 75%) and poor knowledge if they scored below this threshold.

Male partners' attitudes towards child feeding practices were measured through eight questions on a 5‐point Likert scale ranging from 1 (strongly disagree) to 5 (strongly agree). The aggregate attitude score was derived by summing the Likert scale values for all 8 attitude questions, with higher scores indicating more positive attitudes. Participants scoring ≥ the mean were classified as having positive attitudes; those below the mean had negative attitudes (Bogale et al. 2022; Wolkanto et al. 2023).

### 2.6 Study Variables

The outcome variable of interest was male partner involvement in child feeding. On the other hand, 14 independent variables were considered for the study. These were: (1) partner age, (2) mother age, (3) mother's educational status, (4) partner education status, (5) partner occupation, (6) mother occupation, (7) family size, (8) index child age, (9) index child sex, (10) index child birth order, (11) partner knowledge, (12) partner attitude, (13) traditional gender roles, and (14) community discouraging male involvement.

### 2.7 Operational Definitions

Male partner: Biological father or caretaking male in the household with age greater than 18 years old and cohabiting with the child's mother regardless of their marital status.

Male partner involvement: male partners who scored 18 (≥ 80%) and above were considered as having good involvement while below 18 were considered poor involvement (Bogale et al. 2022; Catholic Relief Services 2016).

Knowledge: male partners who correctly answered seven or more of the nine (≥ 75%) knowledge questions were considered as having good knowledge, whereas below seven were considered poor knowledge (Bogale et al. 2022).

Attitude: male partners who scored greater than or equal to the mean were classified as having a positive attitude, whereas those below the mean were labeled as having a negative attitude (Bogale et al. 2022; Wolkanto et al. 2023).

Traditional gender role misconception: The socially ingrained belief that considers child feeding and child‐rearing responsibilities to be exclusively or predominantly the duty of mothers, while marginalizing or excluding the role of fathers.

### 2.8 Data Quality Control

Qualified data collectors and supervisors received comprehensive training on the KoboToolbox system, data collection tool, interview technique, and ethical consideration. The data collection tool was pre‐tested on 5% of the sample (21 male partners) in Adare Kebele, Bahil Adarash sub‐city. Based on results, technical terms were simplified, ambiguous items clarified, redundant questions removed, and skip logic optimized. Rigorous supervision included daily check‐ups of collected data and prompt error corrections. The use of the KoboToolbox system for data collection facilitated logical data entry and maintained data quality. Supervisors verified form completeness, ensuring data integrity throughout the process.

### 2.9 Data Processing and Analysis

Data collection was conducted via KoboToolbox and subsequently transferred to SPSS version 26.0 for processing and analysis. Descriptive statistics, including frequency distributions, central tendency, and dispersion measures, were utilized. Bi‐variable and multivariable logistic regression analyses assessed the association between independent variables and the outcome variable. Variables with a *p*‐value < 0.25 in the bivariable analysis were incorporated into the multivariable model to control for confounding factors (Bursac et al. 2008; Chen and Dey 2003). Model adequacy was evaluated using the Hosmer–Lemeshow test (*p* = 0.899), while multicollinearity was checked through the variance inflation factor (VIF). Statistical significance in the multivariable analysis was set at *p* < 0.05, with results reported as AOR and 95% CI.

## 3 Results

### 3.1 Socio‐Demographic and Economic Characteristics of the Participants

Out of 422 male partners selected for the study, 419 participated, yielding a response rate of 99.3%. The mean (± standard deviation) age of study participants was 33.7 years (± 6.7), with 50.8% falling within the 26–35 age group. One hundred sixty‐four (39.1%) had attained college‐level or higher education, while 40.8% were government employees. Among mothers, 34.4% had completed secondary education, and 43.2% were housewives. Over half (58.9%) of households comprised five or fewer members. Less than one‐fourth (22.9%) of respondents reported a monthly income of ≥ 9001 Ethiopian Birr (160.1 $SD) (Table 1).

**TABLE 1:** Socio‐demographic and economic characteristics of the study participants in Hawassa City Administration, Sidama Region, Ethiopia.

Characteristics (*n* = 419)	Category	Frequency	Percent
Age (years)	≤ 25	55	13.1
	26–35	213	50.8
	≥ 36	151	36.0
Educational status	No formal education	54	12.9
	Primary education	82	19.6
	Secondary education	119	28.4
	College or above	164	39.1
Occupation	Farmer	16	3.8
	Government employee	171	40.8
	Daily laborer	42	10.0
	Merchant	87	20.8
	NGO employee	57	13.6
	Unemployed	46	11.0
Educational status of the mother	No formal education	77	18.4
	Primary education	91	21.7
	Secondary education	144	34.4
	College or above	107	25.5
Occupation of the mother	Housewife	181	43.2
	Government employee	86	20.5
	Daily laborer	36	8.6
	Merchant	70	16.7
	NGO employee	29	6.9
	Other	17	4.1
Monthly income (Birr)	≤ 3000 ($53.3)	77	18.4
	3001–6000 ($53.4–$106.7)	135	32.2
	6001–9000 ($106.8–$160.0)	111	26.5
	≥ 9001 ($160.1)	96	22.9
Household family size	≤ 5	247	58.9
	> 5	172	41.1

### 3.2 Child Related Characteristics

The majority (66.1%) of the participants had two under five children. The mean (SD) age of the index children was 14.8 (± 5.1) months. More than half (53.0%) of the index children were females. Slightly less than one‐fourth (23.4%) of the index children were the first child in the family (Table 2).

**TABLE 2:** Child related characteristics of the study participants in Hawassa City Administration, Sidama Region, Ethiopia.

Characteristics (*n* = 419)	Category	Frequency	Percent
Number of under five children	One child	94	22.4
	Two children	277	66.1
	Three or more	48	11.5
Age of the child (in months)	6–11	128	30.5
	12–23	291	69.5
Sex of the child	Male	197	47.0
	Female	222	53.0
Birth order of the child	First child	98	23.4
	Not first child	321	76.6

### 3.3 Knowledge and Attitude Towards Child Feeding

The mean (SD) knowledge score was 6.5 (± 1.4) and the majority (76.4%) of the participants had good knowledge about child feeding. The mean (SD) attitude score was 5.1 (± 3.5) and about 58.9% of the participants had a positive attitude (Table 3).

**TABLE 3:** Knowledge and attitude of the study participants towards child feeding in Hawassa City Administration, Sidama Region, Ethiopia.

Variable (*n* = 419)	Category	Frequency	Percent (%)
Knowledge	Good	320	76.4
	Poor	99	23.6
Attitude	Positive	247	58.9
	Negative	172	41.1

### 3.4 Culture and Norm Related Factors

One hundred seventy‐eight (42.5%) study participants reported that the community discourages male involvement in child feeding. More than a third of participants (37.2%) reported that the mother did not want their involvement. The majorities (62.1%) stated that the community shames those involved. Less than half (46.8%) revealed that belief in traditional gender‐specific roles and (40.1%) perceived that the community's belief that child feeding is solely the mother's responsibility (Table 4).

**TABLE 4:** Culture and norm related factors of study participants in Hawassa City Administration, Sidama Region, Ethiopia.

Variable (*n* = 419)	Category	Frequency	Percent (%)
Community discourage male involvement	Yes	178	42.5
	No	241	57.5
Mother did not want male involvement	Yes	156	37.2
	No	263	62.8
Community shames male involvement	Yes	260	62.1
	No	159	37.9
Belief traditional gender specific role	Yes	196	46.8
	No	223	53.2
Perceived community belief child feeding is the only responsibility of mothers	Yes	168	40.1
	No	251	59.9

### 3.5 The Magnitude of Male Partner Involvement in Child Feeding Practice

**FIGURE 1:**
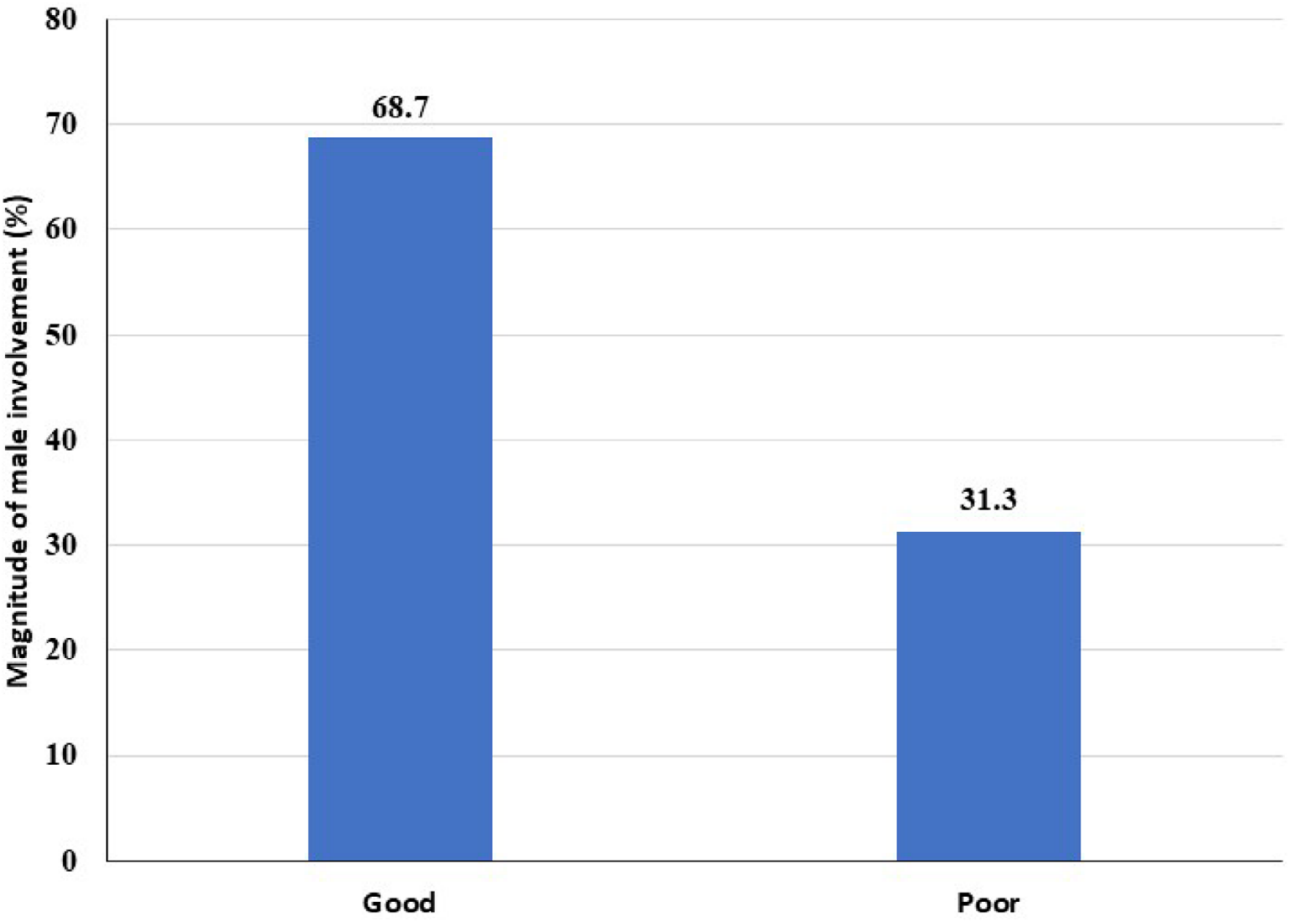
The magnitude of male partner involvement in child feeding practice in households having children aged 6–23 months in Hawassa City, Sidama Region, Ethiopia.

The study presented that the magnitude of good male partners involvement in child feeding practices was 68.7% (95% CI: 64.7%, 72.7%) (Figure 1).

### 3.6 Factors Associated With Male Partner Involvement in Child Feeding Practice

Fourteen variables were included in the bivariable logistic regression analysis. Ten variables with a *p*‐value < 0.25 were selected as candidates for the multivariable model. In the final multivariable analysis, male partner age, educational level, index child age, knowledge, attitudes, and adherence to traditional gender roles misconception emerged as significant predictors of male involvement in child feeding practices.

Male partners aged ≤ 25 and 26–35 years exhibited 2.32 and 2.38 times higher odds, respectively, of being involved in child feeding compared to those aged ≥ 36 years. Male partners without formal education had 72% lower odds of active participation in child feeding than those with a college degree or higher (AOR = 0.28, 95% CI: 0.14–0.59). Male partners with index children aged 6–11 months were nearly twice as likely to show active involvement in child feeding compared to those with children aged 12–23 months (AOR = 1.94, 95% CI: 1.13–3.32).

Male partners with good knowledge of child feeding were twice as likely to participate actively compared to those with limited knowledge (AOR = 2.14, 95% CI: 1.26–3.64). Those with favorable attitudes showed nearly three times higher odds of being actively involved in child feeding than those with unfavorable attitudes (AOR = 2.66, 95% CI: 1.55–4.56). Conversely, male partners who adhered to traditional gender role misconceptions had 59% lower odds of active participation compared to those who did not (AOR = 0.41, 95% CI: 0.24–0.69) (Table 5).

**TABLE 5:** Multivariable analysis of factors associated with male partner involvement in child feeding practice in Hawassa City, Sidama Region, Ethiopia, 2024.

Variables	Male partner involvement		COR (95% CI)	AOR (95% CI)	*p*
	Good (%)	Poor (%)			
Age of male partner (years)					
≤ 25	42 (76.4)	13 (23.6)	2.13 (1.06–4.30)	2.32 (1.07–5.31)*	0.034
26–35	155 (72.8)	58 (27.2)	1.76 (1.13–2.75)	2.38 (1.39–4.09)**	0.002
≥ 36	91 (60.3)	60 (39.7)	1	1	
Age of the mother (years)					
≤ 25	113 (61.1)	72 (38.9)	0.65 (0.26–1.64)	0.43 (0.15–1.21)	0.110
26–35	158 (75.2)	52 (24.8)	1.25 (0.49–3.19)	0.95 (0.35–2.61)	0.927
≥ 36	17 (70.8)	7 (29.2)	1	1	
Educational status of male partner					
No formal education	23 (42.6)	31 (57.4)	0.25 (0.13–0.47)	0.28 (0.14–0.59)**	< 0.001
Primary education	61 (74.4)	21 (25.6)	0.97 (0.53–1.78)	1.15 (0.58–2.29)	0.682
Secondary education	81 (68.)	38 (31.9)	0.71 (0.42–1.19)	0.85 (0.48–1.52)	0.585
College or above	123 (75.0)	41 (25.0)	1	1	
Family size					
≤ 5	162 (65.6)	85 (34.4)	0.69 (0.45–1.07)	0.84 (0.49–1.41)	0.502
> 5	126 (73.3)	46 (26.7)	1	1	
Age of the index child					
6–11	101 (78.9)	27 (21.1)	2.08 (1.28–3.39)	1.94 (1.13–3.32)*	0.016
12–23	187 (64.3)	104 (35.7)	1	1	
Sex of the index child					
Male	142 (72.1)	55 (27.9)	1.34 (0.89–2.04)	1.29 (0.81–2.07)	0.290
Female	146 (65.8)	76 (34.2)	1	1	
Birth order of the index child					
First child	75 (76.5)	23 (23.5)	1.65 (1.05–2.79)	1.49 (0.81–2.72)	0.201
Not first child	213 (66.4)	108 (33.6)	1	1	
Knowledge					
Good	234 (73.1)	86 (26.9)	2.27 (1.42–3.62)	2.14 (1.26–3.64)**	0.005
Poor	54 (54.5)	45 (45.5)	1		
Attitude					
Positive	184 (74.5)	63 (25.5)	1.91 (1.26–2.90)	2.66 (1.55–4.56)**	< 0.001
Negative	104 (60.5)	68 (39.5)	1	1	
Traditional gender role misconception					
Yes	125 (63.8)	71 (36.2)	0.65 (0.43–0.98)	0.41 (0.24–0.69)**	0.001
No	163 (73.1)	60 (26.9)	1	1	

* Significant at *p* < 0.05.

** Significant at *p* < 0.01.

## 4 Discussion

This community‐based study aimed to assess the magnitude of male partner involvement in child feeding and identify associated factors among households with children aged 6–23 months in Hawassa City Administration, Sidama Region, Ethiopia. The study revealed that 68.7% of male partners demonstrated active involvement in child feeding practices. Key factors influencing involvement included the age and education level of male partners, the age of the child, knowledge, attitudes, and prevailing traditional gender role misconceptions.

The findings of this study reveal that the level of male partner engagement in child feeding practices was 68.7%, a figure that aligns closely with results reported from Uganda (65.5%, 68%) (Kansiime et al. 2017; Nyombi and Kirungi 2022). However, lower magnitudes of paternal involvement were documented in studies conducted in Ethiopia (43.1%, 50.9%) (Wolkanto et al. 2023; Bogale et al. 2022), and Ghana (63.5%) (Saaka et al. 2023). In contrast, the current estimate is somewhat lower than those observed in research in India (73%) (Sachdeva and Gupta 2022), and Kenya (80%) (Muthiru 2020). These disparities in the degree of father participation in child feeding can likely be attributed to variations in socioeconomic conditions, cultural norms, methodological approaches, and the demographic profiles of study populations.

The present study identified a statistically significant association between the age of male partners and their level of engagement in child feeding practices. Male partners aged 25 years or younger and those between 26 and 35 years were twice as likely to exhibit higher involvement in child feeding compared to their counterparts aged 36 years and above. This observation is consistent with findings from a study conducted in the Abim District of Uganda (Nyombi and Kirungi 2022). The greater participation among younger fathers may be attributed to their higher levels of education and reduced adherence to traditional cultural norms that often discourage men from taking an active role in childcare (Dinga et al. 2018). Furthermore, younger fathers residing in urban settings, particularly those with fewer children, tend to be more actively involved in child feeding activities (Nyombi and Kirungi 2022).

The level of education among male partners was found to be a significant predictor of their engagement in child feeding practices. Male partners without formal education were 72% less likely to participate in child feeding compared to those with a college degree or higher. This result aligns with studies conducted in India (Sachdeva and Gupta 2022), Nepal (Bhatta 2013), and Ethiopia (Bogale et al. 2022), which similarly reported that fathers with advanced education were more actively involved in child feeding. This trend can be explained by the fact that educated fathers possess greater awareness and more favorable attitudes towards optimal child nutrition and care practices. Furthermore, higher levels of education often foster more equitable views on gender roles, promoting shared responsibility for childcare. Educated fathers are also better equipped to communicate effectively with mothers regarding the nutritional needs of their children.

The findings of this study indicate that male partners with infants aged 6–11 months were twice as likely to exhibit strong participation in child feeding compared to those with children between 12 and 23 months. This trend contrasts with a study conducted in Ghana, which reported greater paternal involvement in feeding among younger children than older ones. (Saaka et al. 2023). The 6–11‐month age range marks a crucial dietary transition, as infants begin shifting from exclusive breastfeeding or formula feeding to the introduction of solid food. This phase necessitates increased parental supervision due to potential risks such as choking, allergic reactions, and the need for a nutritionally adequate diet. This developmental stage provides an important window for father‐infant bonding, potentially enhancing paternal engagement in feeding practices.

The current study revealed that the knowledge level of male partners significantly influenced their active participation in child feeding practices. Male partners with a strong understanding of child nutrition were twice as likely to be highly engaged in complementary feeding compared to those with limited knowledge. This finding is consistent with research conducted in India (Sachdeva and Gupta 2022) and northeast Ethiopia (Bogale et al. 2022). The observed association may stem from the fact that greater knowledge equips fathers to make informed decisions regarding child feeding, enabling them to recognize their vital role in ensuring proper nutrition. Such awareness likely fosters increased involvement in feeding activities, as evidenced by the heightened participation of well‐informed fathers in this study.

Male partners with a favorable outlook on child feeding were found to be three times more likely to actively participate in complementary feeding duties compared to those with less positive attitudes. This result is consistent with study findings from India (Sachdeva and Gupta 2022), Ghana (Saaka et al. 2022), northeast Ethiopia (Bogale et al. 2022), and southern Ethiopia (Wolkanto et al. 2023). This finding may be explained by a behavioral modification in male partners attitudes towards child care and feeding brought on by improved knowledge; men are more active in complementary feeding if they consider it as a shared responsibility rather than just a mother's duty.

The study found that male partners who adhered to traditional gender roles misconceptions were 59% less likely to participate actively in child feeding compared to those who did not subscribe to such beliefs. This result is consistent with research conducted in northeast Ethiopia, which identified a significant link between cultural practices, including adherence to gender‐specific roles, and paternal involvement in child feeding (Bogale et al. 2022). This finding can be explained by the influence of cultural norms and societal expectations. In communities where traditional gender roles are deeply entrenched, men may perceive child feeding as primarily a woman's responsibility, leading to their reduced engagement in such activities.

## 5 Strength and Limitation of the Study

A key strength of this study lies in its focus on male involvement in child feeding practices within an urban Ethiopian setting, a topic that has received limited attention in prior research. By examining multiple dimensions of involvement—such as knowledge, attitudes, decision‐making, and supportive behaviors—the study offers a holistic assessment. However, the cross‐sectional design limits the ability to infer causal relationships between variables. Moreover, reliance on self‐reported data may have introduced social desirability bias, while responses based on past experiences could be subject to recall bias. The exclusion of mothers' perspectives further narrows the scope of the findings.

## 6 Conclusion

The study revealed that the magnitude of good involvement was found to be 68.7%, reflecting relatively high male engagement in child feeding responsibilities in this urban setting. Male partner age, education level, knowledge, attitudes, and traditional gender role misconception impacted the involvement of male partners in Hawassa City Administration, Sidama Region, Ethiopia.

## 7 Recommendation

The Hawassa City Administration Health Department should enhance health education programs to improve fathers' knowledge of optimal child feeding practices and the importance of parental involvement, addressing gender norms that restrict male participation. Urban health extension workers should promote positive attitudes towards shared childcare responsibilities, targeting older fathers. Future studies should use qualitative methods to explore barriers faced by fathers and maternal perspectives on male involvement in child feeding.

## Author Contributions

**Amelo Bolka:** conceptualization (equal), formal analysis (equal), funding acquisition (equal), investigation (equal), methodology (equal), software (equal), supervision (equal), writing – original draft (equal), writing – review and editing (equal). **Mahlet Assaye:** conceptualization (equal), data curation (equal), formal analysis (equal), funding acquisition (equal), investigation (equal), methodology (equal), resources (equal), software (equal), writing – original draft (equal), writing – review and editing (equal). **Assefa Phliphos:** conceptualization (equal), data curation (equal), formal analysis (equal), investigation (equal), methodology (equal), project administration (equal), software (equal), supervision (equal), validation (equal), visualization (equal), writing – review and editing (equal).

## Ethics Statement

This study adheres to the ethical principles outlined in the Declaration of Helsinki. The Institutional Review Board (IRB) of Yirgalem Hospital Medical College granted ethical clearance for this study (Protocol Number‐YHMC/IRB/001, Date‐11/22/2023). Hawassa City Administration Health Department wrote a permission letter to selected kebeles. Written informed consent was obtained from all parents/caretakers of the children following a thorough explanation of the study objective. All data have been anonymized to ensure participant confidentiality.

## Consent

The authors have nothing to report.

## Conflicts of Interest

The authors declare no conflicts of interest.

## Data Availability

The datasets analyzed during the current study are not publicly available due to institutional regulation but are available from the corresponding author on reasonable request.
